# Favourable mid-term isokinetic strength after primary THA combined with a modified enhanced recovery after surgery concept (ERAS) in a single blinded randomized controlled trial

**DOI:** 10.1007/s00402-024-05479-z

**Published:** 2024-08-06

**Authors:** Jan Reinhard, Julia Sabrina Schiegl, Stefano Pagano, Franziska Leiss, Tobias Kappenschneider, Günther Maderbacher, Joachim Grifka, Felix Greimel

**Affiliations:** Department of Orthopedic Surgery, University Medical Center Regensburg, Kaiser-Karl-V-Allee 3, 93077 Bad Abbach, Germany

**Keywords:** Total hip arthroplasty (THA), Fast track surgery, Early mobilization, Enhanced recovery after surgery (ERAS), Biodex, Isokinetic strength measurement, Mid-term outcome

## Abstract

**Purpose:**

Muscular deficits as part of severe osteoarthritis of the hip may persist for up to two years following total hip arthroplasty (THA). No study has evaluated the mid-term benefit of a modified enhanced-recovery-after-surgery (ERAS) concept on muscular strength of the hip in detail thus far. We (1) investigated if a modified ERAS-concept for primary THA improves the mid-term rehabilitation of muscular strength and (2) compared the clinical outcome using validated clinical scores.

**Methods:**

In a prospective, single-blinded, randomized controlled trial we compared patients receiving primary THA with a modified ERAS concept (*n* = 12, ERAS-group) and such receiving conventional THA (*n* = 12, non-ERAS) at three months and one year postoperatively. For assessment of isokinetic muscular strength, a Biodex-Dynamometer was used (peak-torque, total-work, power). The clinical outcome was evaluated by using clinical scores (Patient-Related-Outcome-Measures (PROMs), WOMAC-index (Western-Ontario-and-McMaster-Universities-Osteoarthritis-Index), HHS (Harris-Hip-Score) and EQ-5D-3L-score.

**Results:**

Three-months postoperatively, isokinetic strength (peak-torque, total-work, power) and active range of motion was significantly better in the modified ERAS group. One year postoperatively, the total work for flexion was significantly higher than in the Non-ERAS group, whilst peak-torque and power did not show significant differences. Evaluation of clinical scores revealed excellent results at both time points in both groups. However, we could not detect any significant differences between both groups in respect of the clinical outcome.

**Conclusion:**

With regard to muscular strength, this study supports the implementation of an ERAS concept for primary THA. The combination with a modified ERAS concept lead to faster rehabilitation for up to one-year postoperatively, reflected by significant higher muscular strength (peak-torque, total-work, power). Possibly, because common scores are not sensitive enough, the results are not reflected in the clinical outcome. Further larger randomized controlled trials are necessary for long-term evaluation.

**Supplementary Information:**

The online version contains supplementary material available at 10.1007/s00402-024-05479-z.

## Introduction

Enhanced recovery after surgery concepts (ERAS) have recently experienced growing acceptance in orthopaedic surgery [1–3]. Being initially established for colorectal surgery, a number of studies proved less adverse reactions and reduced morbidity [1, 4–8]. Primary total hip arthroplasty (THA) is one of the most frequent and most successful operations in the world. The number of primary THA is rapidly increasing [9]. Recent projections predict an increase of primary THA by 659% in 2060 in the U.S., reaching 1,982,099 operations annually [10]. Even though primary THA is one the most successful operations worldwide, about 10% of patients report postoperative dissatisfaction [11, 12]. The main reason being muscular insufficiencies, mainly of gluteus medius and minimus [13]. Preoperative existing muscular deficiencies as part of severe osteoarthritis of the hip may persist for up to two years postoperatively after primary THA [9, 14, 15]. Enhanced recovery after surgery concepts with early mobilization aim to counteract the postoperative pathophysiologic catabolism and therefore aim to improve the muscular strength. However, no study performed a mid-term isokinetic strength measurement after primary THA in combination with a modified ERAS concept in detail. To assess patients’ muscular strength, isokinetic dynamometers such as the Biodex system represent the gold standard [8]. Most isokinetic strength measurement studies focus on the knee joint. In contrast, isokinetic strength measurement of the hip is rarely performed and therefore strong reference values are currently missing [16].

### Aim of the study

This prospective randomized controlled trial (1) investigates if a modified ERAS-Concept for primary THA improves the mid-term rehabilitation of muscular strength at three months and one year postoperatively in comparison to conventional THA. Furthermore, both groups (2) were compared in terms of the patient-related outcome measures (PROMs) and validated clinical scores (HHS (Harris hip score), WOMAC index (Western Ontario and McMaster Universities Osteoarthritis Index), and EQ-5D-3L).

## Methods

The data assessment took place between 01/04/2021 and 01/02/2023 at the orthopaedic department of a German university hospital. This study demonstrates the follow-up data of the initial study “Comparison of postoperative isokinetic Quadriceps and Gluteal muscular strength after primary THA: Is there an early benefit through Enhanced recovery programs?” published in 2023 [17]. The present study is part of a large single-blinded randomized controlled trial, which started in 2019 and compares a modified ERAS concept for primary THA with a conventional (Non-ERAS) concept [18].

The major criteria for inclusion was medical signs of primary or secondary hip osteoarthritis and indication for primary THA. The age span was set at above 18 and under 90 years. Patients with extremely reduced mobility (walking distance less than 100 m, permanent use of a walker / wheelchair), having had earlier surgical interventions on the same hip, showing a body mass index (BMI) above 40 kg/m^2^ or suffering from a skeletal tumoral disease or having pronounced muscular contractures were excluded from the study. Patients who fulfilled these criteria were enrolled during the consultation hour. Participation was voluntarily and withdrawal was possible at any time. Patients were randomized on both groups by using closed envelopes. Three senior orthopaedic surgeons performed primary THA. Only the surgeons, who were not involved in the follow-up readmissions, were unblinded due to regulatory issues in Germany (use of medication intraoperatively in the ERAS group). The rest of the study team as well as the patients were blinded. Postoperatively, the participants of each concept were treated on different wards to prevent unblinding and a possible bias.

The study was conducted in agreement with the ethical standards of the Declaration of Helsinki (1975). It was approved by the local Ethics Committee (approval number 19-1308-101) and is registered in the DRKS (DRKS00031345, WHO register).

### Similarities of both concepts

The patients in both groups underwent the same operation. They received primary THA, using a modified Watson-Jones approach without transection of muscular tissue. The advantage of this approach lies in the preserved integrity of the surrounding muscles and posterior capsule which prevents posterior dislocation. Cementless implants were used. The pain management concept was based upon the three step analgesic ladder, established by the World health organization (WHO). All participants received Ibuprofen and Metamizole as basic pain medication, depending on allergies and further diseases. In addition, patients within the modified ERAS group received 10/5 mg oxycodone/naloxone once on the day of surgery. Additional pain medication was applied depending on the subjective patient rating using the numeric rating scale (NRS). Patients received 3 mg piritramide optionally on the intermediate care unit. On the ward, patients were allowed to have 100 mg tramadol or 10/5 mg oxycodone/naloxone as additional “rescue medication”.

In line with the general laws of the German health care system (SGBV) and to maintain standardized study group comparison, patients of both groups were discharged to the rehabilitation clinic seven days after surgery. All patients in the rehabilitation clinic received the same therapy for three weeks. Following discharge, they generally returned to their daily routine. The time points for readmission were chosen by looking at the expected progress with regard to the return to the individual daily routine, which was only lightly affected three months after surgery and not affected after one year.

### The modified enhanced recovery after surgery concept (ERAS)

Every patient in the modified enhanced recovery after surgery group (ERAS group) received preoperative gait training with crutches and was educated on the precautions after THA and pain management. One hour preoperatively, patients received a single dose of non-steroid-anti-inflammatory analgesia (etoricoxibe 90 mg). A short-lasting spinal anaesthesia (4 ml prilocaine 1%, hyperbaric and 10 µg sufentanyl) in combination with intravenously administration of dexamethasone (8 mg) was chosen. Tranexamic acid was applied systemically (1 g) and topically (2 g). In addition, local-infiltration analgesia (ropivacaine 200 mg, adrenaline 0.5 mg) was performed intraoperatively. No drains were inserted. Patients in the modified ERAS group were first mobilized two to three hours after surgery and full weight-bearing was allowed immediately following the surgery. For the first mobilization, a walking distance of 50 m was targeted. In a multidisciplinary team, a special physiotherapy treatment protocol was developed. Specially trained physiotherapists performed targeted physiotherapy twice a day. Patients were instructed to practice independently on a special exercise circuit. The exercise circuit combined different workouts for muscle formation, a walking course and coordination tasks.

### The conventional setup (Non-ERAS)

Patients who underwent primary THA combined with a conventional setup (non-ERAS) received neither the special patient education described above, nor the single dose analgesics preoperatively. Anaesthesia was conducted by a long-lasting spinal anaesthesia (4 ml bupivacaine, 0.5% and fentanyl). Intraoperatively, neither local infiltration analgesia nor tranexamic acid was administered. Wound drains were inserted in every patient. First mobilization was performed on the first day after surgery. As in the ERAS group, patients were allowed full weightbearing immediately after surgery. Physiotherapy was conducted once a day and patients did not use the exercise circuit. (Table 1).

**Table 1:** Comparison of the two concepts for primary THA.

	Non-ERAS (*n* = 12)	Modified ERAS (*n* = 12)
**preoperatively**		
gait training with crutches	**-**	**+**
patient education	**-**	**+**
etoricoxibe 90 mg p.o. preoperatively	**-**	**+**
**intraoperatively**		
Cementless implants	**+**	**+**
short lasting spinal anesthesia (Prilocain 1%, 10 µg sufentanil) Dexamethasone 8 mg i.v	**-**	**+**
long-lasting spinal anesthesia (4 ml bupivacaine 0.5% and fentanil)	**+**	**-**
local infiltration analgesia (periacetabular, femoral, subcutaneously)	**-**	**+**
tranexamic acid local and topically	**-**	**+**
drains	**+**	**-**
**postoperatively**		
first mobilization	1 d postoperatively	2–3 h postoperatively
full weight bearing	**+**	**+**
physiotherapy first week	1x/d	2x/d
exercise circuit first week	**-**	**+**
rehabilitation clinic for three weeks	**+**	**+**

### Clinical examination, PROMs, WOMAC, HHS, EQ-5D-3L

At every consultation, patients were interviewed about pain intensity, quantified by an NRS (zero (no pain) to ten (worst pain)). Additionally, passive range of motion of the operated hip was imposed. Patients were tested for Trendelenburg’s sign and for the ability of carrying out a one-leg standi on the injured side for longer than 15 s. Special patient related outcome measures (PROMs) were used to analyse postoperative quality of life and satisfaction. Furthermore, Harris-Hip-Score (HHS), Western-Ontario-and-McMaster-Universities-Osteoarthritis-Index (WOMAC), were imposed, representing validated scoring tools with a good reliability [19]. The EQ-5D-3L, evaluating the health-related quality of life, was also imposed.

### Isokinetic strength measurement

The technical assessment of isokinetic muscular strength one week preoperatively, as well as three months and one year postoperatively was identical in both groups. A *Biodex System 4 Pro Dynamometer* (Biodex Medical systems, Shirley, NY, U. S.) was used to perform the measurements. In the publication of the short-term results, the measurement protocol is described in detail [17]. The measurements were conducted by two blinded observers. To reach an experimental setup which resembles physiological daily motion, participants were placed in an upright position. The individual adjustment of the Biodex Dynamometer for each patient was maintained for every measurement to gain comparable results. We aimed to measure the most important moving directions of the hip for daily living: Flexion / Extension and Abduction / Adduction. To warm up, patients had to walk a fixed distance of 100 m and they executed five contractions in exercise mode before the final measurement.

Isokinetic strength measurement of Flexion / Extension was done with an angular speed of 60 °/s. For Abduction / Adduction an angular speed of 30 °/s was used. First, the active range of motion was imposed. Following three practice runs using the whole range of motion, the final measurement was performed with maximum strength and five repetitions. Patients were motivated constantly, to achieve and maintain the maximum strength. The fluent and clear execution of each movement was supervised by a study member. In case of evasive movements, the whole measurement was repeated. For every moving direction (Flexion / Extension, Abduction / Adduction), peak torque (Newton meter, Nm), overall work (Joule, J), power (Watt, W) and active range of motion was measured.

### Statistical analysis

The Shapiro-Wilk-Normality-Test was used to test for normal distribution. Metric variables were noted as mean ± standard deviation (SD) if the data is normally distributed or as median ± interquartile range (IQR) if not. Categorical variables were noted in relative frequency. To test for statistical significance, we used the *t-test* if data was normally distributed, or *nonparametric Mann-Whitney U*. Statistical significance was considered *p* < 0.05. Statistical analysis was performed with SPSS (IBM SPSS Statistics 28, International Business Machines Corporation (IBM), Armonk, New York, U.S.).

## Results

**Fig. 1:**
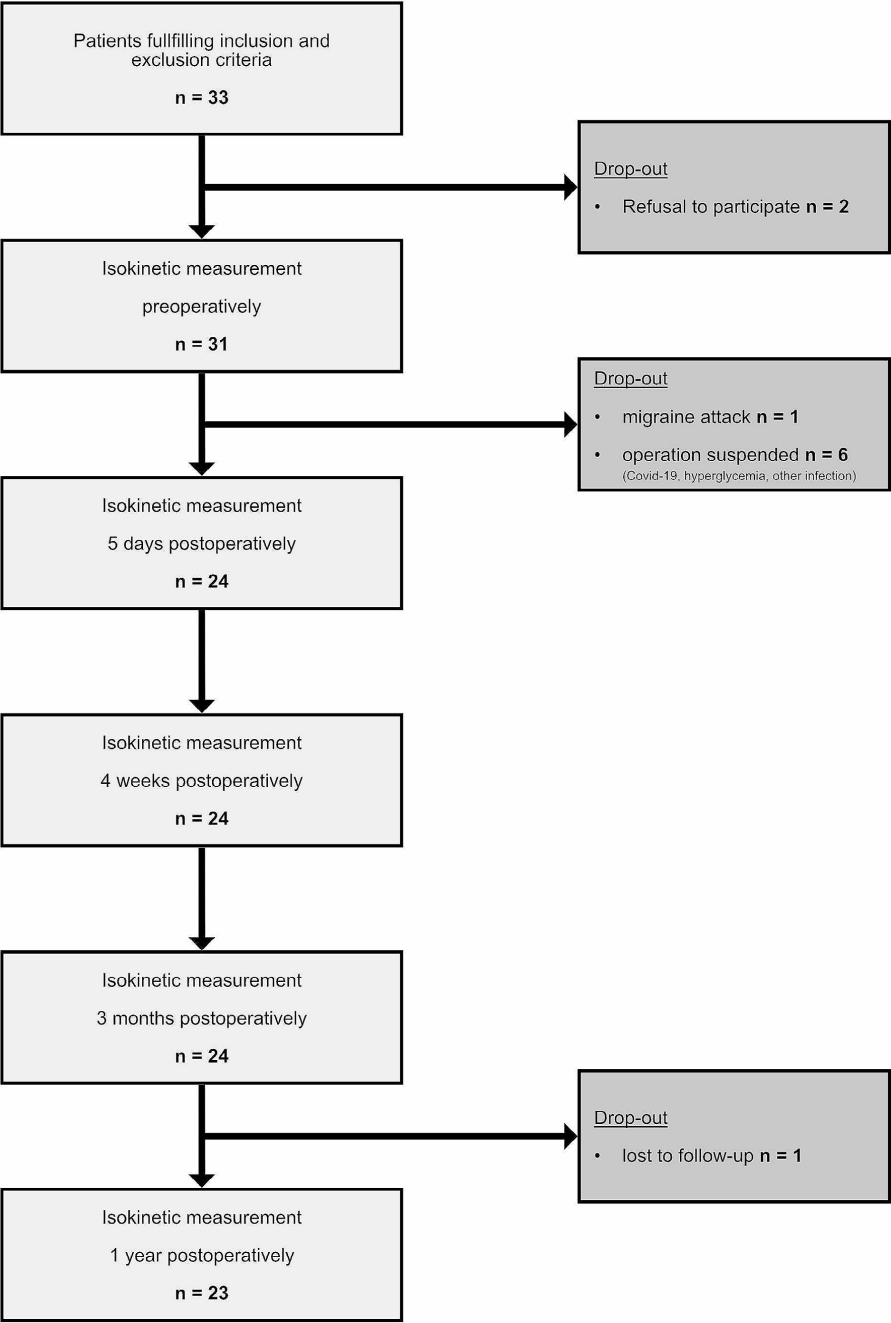
Flowchart enrollment process. Flowchart of the enrollment process and follow-up

Preoperative isokinetic strength measurement was performed in 31 patients. Following preoperative routine diagnostics, three patients were diagnosed with a SARS-Covid-19 infection and the operation was suspended. Two patients showed elevated inflammation values, requiring further diagnostic examination and another patient showed unknown hyperglycemia requiring an amendment and therapy. None of these patients were randomized at that point. Postoperatively, one patient had a severe migraine attack which hindered the measurements and led to exclusion. Altogether, 24 patients were included in the final study (modified ERAS *n* = 12, Non-ERAS *n* = 12). One patient in the Non-ERAS group was lost to follow-up for the one-year readmission. (Fig. 1).

### Demographic data

In terms of demographic data, no significant differences were found between the two groups (*p* > 0.05). (Table 2).

**Table 2:** Demographic data of the 24 included patients

	Non-ERAS (*n* = 12)				Modified ERAS (*n* = 12)				statistical difference
	Mean ± SD		Range		Mean ± SD		Range		p-value
Age (years)	69.75 ± 8.63		55–83		70.0 ± 10.61		52–88		0.97
Sex (male : female)	5 : 7				6 : 6				0.99
Body mass index (BMI)	28.03 ± 5.03		19.83–38.42		27.8 ± 4.18		22.04–36.76		0.89
Injured leg (right : left)	6 : 6				6 : 6				0.99
Dominant leg (right : left)	10 : 2				12 : 0				0.48
Osteoarthritis contralateral (mild / symptomatic)	2 / 1				4 / 0				0.64
Total hip arthroplasty (THA) contralateral	2				1				0.99
ASA Score frequency (relative frequency (%), absolute frequency)	1	2	3	4	1	2	3	4	0.14
	8.3% (1/12)	83.3% (10/12)	8.3% (1/12)	0% (0/12)	33.3% (4/12)	58.3% (7/12)	8.3% (1/12)	0% (0/12)	
Duration of surgery (min)	57.25 ± 13.75		35–85		58.33 ± 12.14		36–77		0.28

### Functional outcome of the operated hip

Both groups showed comparable values of Trendelenburg’s sign and the ability to perform a one-leg stand at all time points, showing excellent results at the one-year time point. We could not detect any significant differences between both groups at any time point (*p* > 0.05). Overall, patients in the ERAS group showed a tendency towards an improved passive range of motion. We detected a significant increased passive range of motion for flexion at three months in the modified ERAS group (*p* = 0.04). (Table 3).

**Table 3:** Functional outcome of the operated hip

		Non-ERAS (*n* = 12)	Modified ERAS (*n* = 12)	statistical difference
**positive Trendelenburg’s sign (relative frequency (%)**,** absolute frequency)**				p-value
PRE-OP		16.7 (2/12)	25 (3/12)	0.99
3 m POST-OP		0 (0/12)	0 (0/12)	0.99
1 y POST-OP		0 (0/11)	0 (0/12)	0.99
**ability to perform one-leg stand > 15s (relative frequency (%)**,** absolute frequency)**				
PRE-OP		66.7 (8/12)	58.3 (7/12)	0.99
3 m POST-OP		91.7 (11/12)	91.7 (11/12)	0.99
1 y POST-OP		100 (11/11)	100 (12/12)	0.99
**passive range of motion (°)**,** mean ± SD**				
PRE-OP	Flexion	103.1 ± 14.9	95.5 ± 7.6	0.36
	Abduction	26.3 ± 5.2	21.5 ± 6.7	0.12
3 m POST-OP	Flexion	103.3 ± 12.3	113.3 ± 11.6	***0.04***
	Abduction	37.5 ± 12.2	39.2 ± 7.9	0.49
1 y POST-OP	Flexion	107 ± 14.2	115.2 ± 11.7	0.13
	Abduction	39.1 ± 8.3	41.7 ± 5.8	0.3

### Patient related outcome measures (PROMs)

In both groups, the patient related outcome measures showed great patient satisfaction at three months and one year postoperatively. We could not detect any significant differences between both groups at any time point. (Table 4).

**Table 4:** Results of patient related outcome measures (PROMs)

		Non-ERAS (*n* = 12)		Modified ERAS (*n* = 12)		statistical difference (*p*-value)	
PROMS (absolute frequencies)		3 m POST-OP	1 y POST-OP	3 m POST-OP	1 y POST-OP	3 m POST-OP	1 y POST-OP
How do you rate the function of your hip?	Normal	10/12	9/11	6/12	9/12	0.15	0.99
	almost normal	2/12	2/11	5/12	3/12		
	impaired	0/12	0/11	1/12	0/12		
	strongly impaired	0/12	0/11	0/12	0/12		
Do you judge the operation as successful?	yes	12/12	11/11	12/12	12/12	0.99	0.99
	no	0/12	0/11	0/12	0/12		
Would you undergo the operation (THA) again?	yes	12/12	11/11	12/12	12/12	0.99	0.99
	no	0/12	0/11	0/12	0/12		
Have your expectations to the operation been fulfilled?	very strong	7/12	9/11	7/12	8/12	0.99	0.55
	strong	4/12	2/11	3/12	3/12		
	moderate	1/12	0/11	2/12	1/12		
	light	0/12	0/11	0/12	0/12		
	no	0/12	0/11	0/12	0/12		
How do you feel in comparison to your preoperative health condition?	much better	11/12	11/11	9/12	9/12	0.59	0.22
	better	0/12	0/11	3/12	3/12		
	same	1/12	0/11	0/12	0/12		
	worse	0/12	0/11	0/12	0/12		
	much worse	0/12	0/11	0/12	0/12		
Has your quality of life improved?	very strong	8/12	8/11	6/12	8/12	0.37	0.86
	strong	4/12	3/11	4/12	3/12		
	moderate	0/12	0/11	2/12	1/12		
	light	0/12	0/11	0/12	0/12		
	no	0/12	0/11	0/12	0/12		

### WOMAC, HHS, EQ-5D-3L

The WOMAC-index and HHS showed significant better results in total and all sub scores at both time points. The modified ERAS and Non-ERAS group showed comparable values at all time points. We could not detect any significant differences at any time point (*p* > 0.05). The EQ-5D-3L showed comparable results, based on improvement in every category. In conformity with WOMAC and HHS, there were no significant differences between both groups at any time point. (Table 5).

**Table 5:** Western Ontario and McMaster universities osteoarthritis Index (WOMAC), Harris hip score (HHS) and EQ-5D-3L - preoperative, three months and one year postoperatively

		Non-ERAS (*n* = 12)	Modified ERAS (*n* = 12)	statistical difference
WOMAC (mean ± SD)				p-value
Stiffness (0–8)	PRE-OP	3.3 ± 1.8	4.4 ± 1.2	0.18
	3 m POST-OP	0.9 ± 1.5	1.5 ± 1.7	0.36
	1 y POST-OP	0.4 ± 0.8	1.0 ± 1.8	0.48
Pain (0–20)	PRE-OP	8.8 ± 4.1	10.1 ± 3	0.34
	3 m POST-OP	0.6 ± 1.2	1.8 ± 2.3	0.15
	1 y POST-OP	0.2 ± 0.4	0.9 ± 1.6	0.3
Physical Function (0–68)	PRE-OP	28.7 ± 9.8	36.3 ± 8.8	0.08
	3 m POST-OP	4.9 ± 7.8	8.4 ± 12.5	0.83
	1 y POST-OP	1.4 ± 1.6	3.6 ± 5.1	0.66
Total score (0–96)	PRE-OP	40.8 ± 14.1	49.5 ± 14.1	0.12
	3 m POST-OP	6.3 ± 10.0	11.0 ± 15.2	0.78
	1 y POST-OP	1.9 ± 1.8	5.5 ± 8.0	0.91
HHS (mean ± SD)				
pain (0–44)	PRE-OP	17.1 ± 4.9	6.4 ± 1.9	0.55
	3 m POST-OP	40.0 ± 6.6	37.5 ± 8.0	0.62
	1 y POST-OP	42.9 ± 1.9	39.8 ± 7.5	0.42
walking (0–33)	PRE-OP	20.6 ± 8.8	23.1 ± 6.4	0.67
	3 m POST-OP	31.8 ± 2.0	31.8 ± 2.0	0.99
	1 y POST-OP	32.5 ± 1.8	31.5 ± 2.7	0.28
ADL (0–14)	PRE-OP	9.7 ± 2.6	7.8 ± 3	0.14
	3 m POST-OP	12.1 ± 1.6	13.3 ± 1.3	0.63
	1 y POST-OP	13.2 ± 1.4	13.3 ± 1.6	0.7
Total score (0–91)	PRE-OP	47.4 ± 9.8	47.3 ± 11.8	0.99
	3 m POST-OP	83.8 ± 8.0	82.6 ± 10.6	0.78
	1 y POST-OP	88.6 ± 2.4	84.6 ± 11.3	0.94
EQ-5D-3L (mean ± SD)				
flexibility (1–3)	PRE-OP	1.8 ± 0.6	1.7 ± 0.5	0.99
	3 m POST-OP	1.0 ± 0.0	1.2 ± 0.4	0.48
	1 y POST-OP	1.0 ± 0.0	1.1 ± 0.3	0.99
self-supply (1–3)	PRE-OP	1.2 ± 0.6	1.2 ± 0.4	0.99
	3 m POST-OP	1.0 ± 0.0	1.1 ± 0.3	0.99
	1 y POST-OP	1.0 ± 0.0	1.1 ± 0.3	0.99
general tasks (1–3)	PRE-OP	1.7 ± 0.7	1.8 ± 0.4	0.51
	3 m POST-OP	1.0 ± 0.0	1.2 ± 0.4	0.48
	1 y POST-OP	1.0 ± 0.0	1.1 ± 0.3	0.99
pain (1–3)	PRE-OP	2.1 ± 0.3	2.2 ± 0.4	0.99
	3 m POST-OP	1.3 ± 0.5	1.3 ± 0.5	0.99
	1 y POST-OP	1.1 ± 0.3	1.3 ± 0.5	0.32
anxiety (1–3)	PRE-OP	1.3 ± 0.5	1.1 ± 0.3	0.31
	3 m POST-OP	1.1 ± 0.3	1.1 ± 0.3	0.99
	1 y POST-OP	1.1 ± 0.3	1.1 ± 0.3	0.99

### Isokinetic strength measurement

**Fig. 2:**
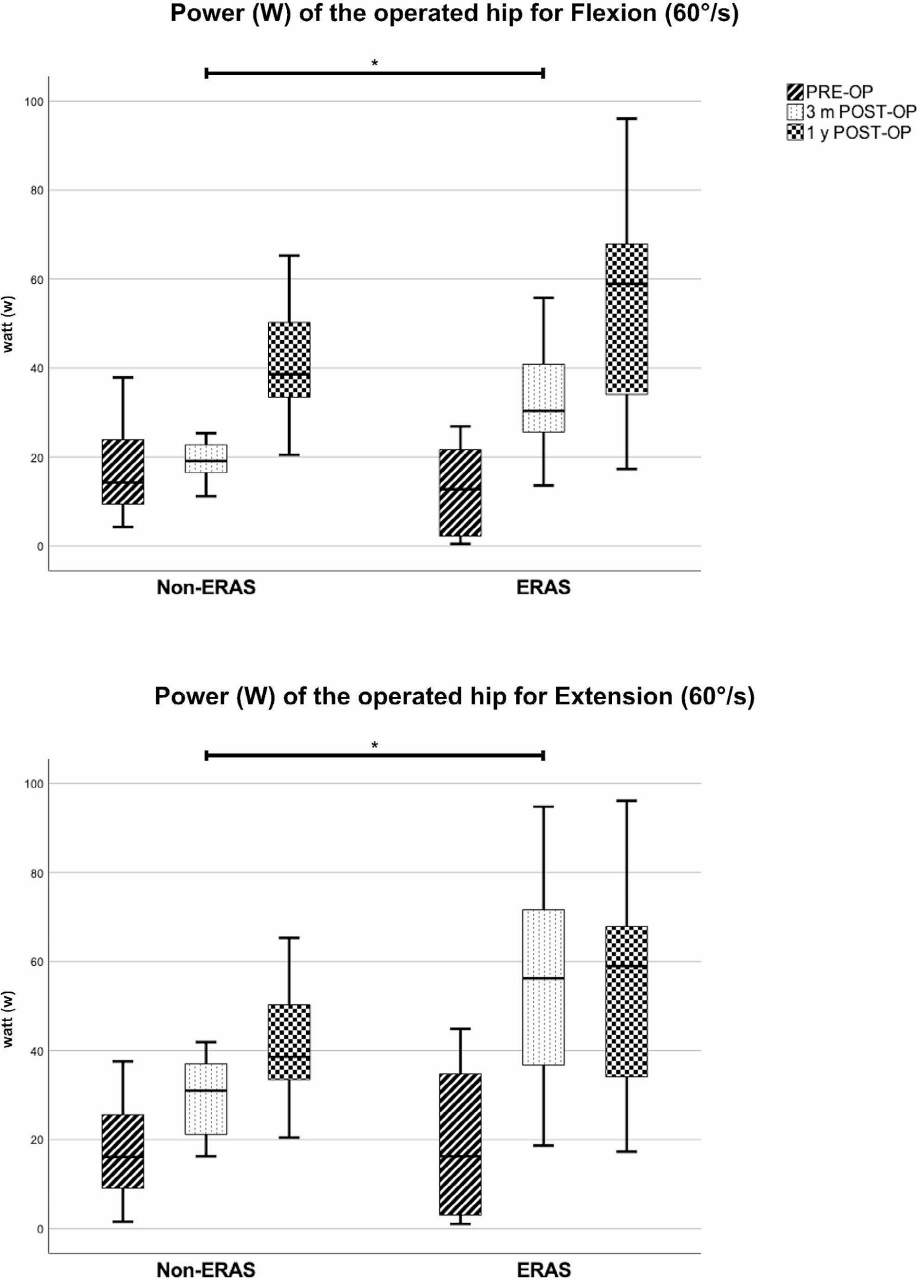
Isokinetic strength measurement – Power (W) analysis for Flexion and Extension (60°/s) on the operated hip, Boxplot. Significant differences between the two groups are marked by *. The y axis represents the power, measured in Watt (w)

**Fig. 3:**
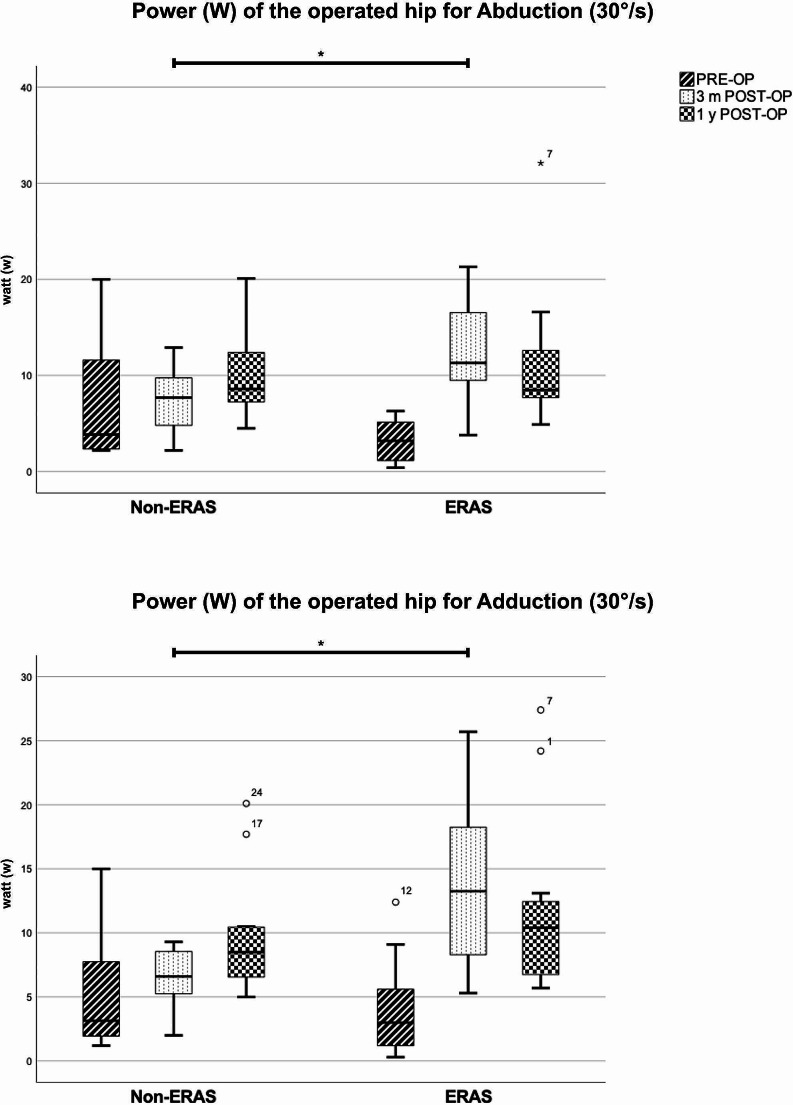
Isokinetic strength measurement – Power (W) analysis for Abduction and Adduction (30°/s) on the operated hip, Boxplot. Significant differences between the two groups are marked by *. The y axis represents the power, measured in Watt (w)

The preoperative isokinetic strength measurement did not show any significant differences between both groups (*p* > 0.05). Only the preoperative active range of motion of abduction, measured by the Biodex system, was significant reduced in the modified ERAS group (*p* = 0.028). The modified ERAS group revealed significant better results for all imposed parameters (peak torque, overall work, power and active range of motion) and all imposed directions of motion (flexion / extension, abduction / adduction) at three months postoperatively (*p* < 0.05). At one year postoperatively we detected a tendency towards better results in the modified ERAS group. The modified ERAS group reached significant greater overall work for flexion (*p* = 0.04). All other parameters did not show significant differences between both groups at one-year postoperatively (*p* > 0.05). (Table 6; Figs. 2 and 3).

**Table 6:** Results of isokinetic strength measurement of the operated hip

		Non-ERAS (*n* = 12)	Modified ERAS (*n* = 12)	statistical difference
peak torque (Nm), mean ± SD				p-value
60°/s flexion	PRE-OP	38.63 ± 18.53	29.13 ± 17.51	0.21
	3 m POST-OP	40.81 ± 8.78	66.03 ± 19.26	***< 0.001***
	1 y POST-OP	67.34 ± 13.65	70.85 ± 23.72	0.08
60°/s extension	PRE-OP	40.22 ± 18.87	41.01 ± 29.67	0.94
	3 m POST-OP	58.37 ± 13.19	103.9 ± 45.62	***0.004***
	1 y POST-OP	76.23 ± 24.03	98.18 ± 36.41	0.13
30°/s abduction	PRE-OP	33.08 ± 18.92	19.06 ± 9.47	0.16
	3 m POST-OP	31.33 ± 10.41	50.23 ± 18.15	***0.004***
	1 y POST-OP	38.97 ± 16.97	46.53 ± 23.01	0.28
30°/s adduction	PRE-OP	25.42 ± 15.79	20.52 ± 14.45	0.48
	3 m POST-OP	28.34 ± 8.18	54.38 ± 21.53	***0.003***
	1 y POST-OP	41.33 ± 17.21	45.95 ± 19.34	0.53
overall work (J), mean ± SD				
60°/s flexion	PRE-OP	144.51 ± 97.26	108.38 ± 85.25	0.34
	3 m POST-OP	141.98 ± 31.22	300.80 ± 114.54	***< 0.001***
	1 y POST-OP	228.12 ± 74.39	315.38 ± 120.33	***0.04***
60°/s extension	PRE-OP	179.43 ± 104.25	171.13 ± 157.67	0.99
	3 m POST-OP	239.70 ± 78.46	524.73 ± 209.99	***0.001***
	1 y POST-OP	379.26 ± 154.37	515.13 ± 223.27	0.13
30°/s abduction	PRE-OP	49.56 ± 43.71	21.13 ± 15.94	0.11
	3 m POST-OP	49.67 ± 23.69	96.21 ± 38.63	***0.003***
	1 y POST-OP	76.95 ± 38.77	85.11 ± 54.28	0.83
30°/s adduction	PRE-OP	39.18 ± 32.45	28.44 ± 29.44	0.24
	3 m POST-OP	44.40 ± 18.25	107.93 ± 58.20	***0.005***
	1 y POST-OP	76.31 ± 42.76	89.10 ± 58.87	0.7
power (Watt), mean ± SD				
60°/s flexion	PRE-OP	16.32 ± 9.78	13.44 ± 10	0.48
	3 m POST-OP	19.33 ± 4.25	32.74 ± 11.42	***< 0.001***
	1 y POST-OP	26.8 ± 7.97	35.41 ± 13.27	0.09
60°/s extension	PRE-OP	17.34 ± 10.38	18.8 ± 16.74	0.99
	3 m POST-OP	29.47 ± 9.16	54.43 ± 22.97	***0.005***
	1 y POST-OP	40.99 ± 13.78	52.78 ± 23.43	0.17
30°/s abduction	PRE-OP	7.06 ± 6.02	3.13 ± 2.12	0.11
	3 m POST-OP	7.31 ± 3.17	12.58 ± 4.99	***0.007***
	1 y POST-OP	10.04 ± 4.70	11.32 ± 7.26	0.77
30°/s adduction	PRE-OP	4.98 ± 4.19	3.93 ± 3.78	0.45
	3 m POST-OP	6.69 ± 2.24	14.29 ± 6.76	***0.002***
	1 y POST-OP	9.86 ± 4.82	11.79 ± 7.01	0.48
active range of motion (°), mean ± SD				
Flexion / Extension	PRE-OP	94.23 ± 19.29	83.89 ± 28.01	0.30
	3 m POST-OP	79.53 ± 8.45	103.76 ± 13.58	***< 0.001***
	1 y POST-OP	95.45 ± 10.71	101.43 ± 8.64	0.17
Abduction / Adduction	PRE-OP	36.23 ± 7.74	27.53 ± 10.22	***0.028***
	3 m POST-OP	34.76 ± 3.58	40.71 ± 6.56	***0.013***
	1 y POST-OP	39.84 ± 5.37	38.52 ± 7.55	0.44

## Discussion

In a prospective, single-blinded randomized controlled trial, we aimed to compare mid-term isokinetic strength in patients who underwent primary THA in combination with a modified ERAS concept, and such who received conventional primary THA and post-treatment. Although muscular deficits as part of severe osteoarthritis of the hip may persist for up to two years following primary THA, this study is the first to evaluate the mid-term benefits of a modified ERAS concept on hip strength in detail.

The patients who received primary THA in combination with a modified ERAS concept showed significant improved muscular strength for up to one year postoperatively. All imposed isokinetic strength parameters at three-months revealed significant better results in the modified ERAS group. In addition, patients in the modified ERAS group had a significant higher active range of motion in all relevant directions. One year postoperatively, patients in the modified ERAS group showed a significant higher overall work in terms of flexion, accompanied by a tendency for better results in all other parameters. While we proved significant differences in isokinetic strength between both groups three-months postoperatively, the differences began to diminish one year after surgery. Osteoarthritis of the hip leads to a loss of muscular strength on the affected side [39–41]. In older patients, the risk for an additional muscle breakdown following immobilization is relatively high. Particularly this cohort may benefit from an ERAS concept. Although the interventions of the modified ERAS concept only took place for up to seven days postoperatively, the benefit was sustained for up to one year. This observation highlights the important role of the first postoperative week and intense early postoperative mobilization, the key focus of ERAS concepts. The modified ERAS concept effectively improved the muscular strength of the hip after primary THA and patients benefitted for up to one year postoperatively. In contrast to many other studies in respect of ERAS concepts, the present study did not focus on a reduction of the length of hospital stay [4].

Isokinetic dynamometers, like the Biodex system that was used, demonstrate the gold standard for a detailed assessment of muscular strength [20]. In contrast, hand-held dynamometers which were used in many other studies are influenced by testers’ strength and sex and show a weak interobserver reliability [21, 22]. The Biodex system is proven a good to excellent test-retest reliability in different studies [20, 23–25]. However, strong reference values for isokinetic strength measurement of the hip are still missing [16]. To achieve an experimental setup as close as possible to daily living, we chose an upright position for the measurements, which has been validated in different studies [26, 27]. Only a few studies measured isokinetic strength in patients with THA. Many focus on the comparison of different approaches to the hip and the resulting muscular strength [28, 29]. Other studies which perform a strength measurement after THA focus on different postoperative training and rehabilitation protocols [9, 30–32]. Beck et al. compared primary THA with an intensive training for one year postoperatively with a control group. They did not detect a significant difference at first measurement at six months, and neither at one year postoperatively [33]. Even though they performed the postoperative training for up to one year, this observation is in line with our data. The differences between both groups seem to diminish over a longer period. In addition, solely intensive physiotherapy appears not be sufficient and a multimodal therapy as an ERAS concept is necessary. In summary, the results implicate a faster postoperative rehabilitation after primary THA with an ERAS concept. The data supports the use of a modified ERAS concept for primary THA.

We detected better results for Trendelenburg’s sign and patients’ ability to perform a one leg stand for more than 15 s at five-days postoperatively. However, three months and one year after surgery these differences diminished. In contrast to the significant higher isokinetic strength in the modified ERAS group three months postoperatively, this was not reflected by the functional tests.

Consistent with the short-term results, the evaluation of the patient related outcome measures (PROMS), the WOMAC index, HHS and EQ-5d-3L did not show significant differences between the two groups at any time point. Both groups achieved excellent results in terms of the clinical outcome at three months and at one year postoperatively, as demonstrated in the WOMAC index and the HHS score. The health-related quality of life, assessed by the EQ-5d-3L, showed great results at both time points. In accordance with the results of the functional tests, the significant lesser improvement of muscular strength in the Non-ERAS group for up to one year postoperatively does not appear to be noticed by the patient himself. This leads to the assumption that the difference in mid-term isokinetic strength is not big enough to be of clinical relevance. However, primary THA belongs to the procedures with the highest postoperative patient satisfaction, which might mask smaller improvements in a smaller cohort. Moreover, the existing clinical scores are not sensitive enough to detect smaller differences. This is in line with our observation, showing an excellent postoperative outcome in both groups according to clinical scores. Some studies report a decline in physical function and health-related quality of life from one-year postoperatively onwards [34–36]. We could not detect a tendency towards such observation one year after surgery neither for isokinetic strength nor for the clinical scores. However, this needs to be addressed by larger long-term randomized controlled trials.

### Limitations and strengths

The main limitation is the small sample size, featuring twelve patients of each group. We were not able to enrol more patients because of the time-consuming measurement procedure, required by the Biodex system. This might be the major reason for the comparable relatively small patient cohorts in most studies assessing isokinetic strength. The measurement procedure requires significant preparation and demonstrates a significant stress for the patient himself as well as for the study team. Although the Biodex system demonstrates the gold standard with a high reliability, the strength measurement still depends on patients’ motivation, pain, and condition on that particular day. It is of utmost importance to maintain the same parameters for each patient throughout the whole study to maximize the comparability. The initial enrolment process featured a high dropout rate of 22.5%. Therefore, the intended number of 30 participants was missed. SARS-CoV-19 infection caused half of the drop-out rate. During the SARS-CoV-19 pandemic, elective surgery was stopped for an extended period. Three patients were excluded from the study because of a newly diagnosed SARS-CoV-19 infection during preoperative testing. In those cases, the operation was suspended for at least twelve weeks. One year postoperatively, one patient was unable to attend her follow-up, because she moved to another city and was not available for examination anymore. (Fig. 1). Strengths are the prospective, single-blinded randomized controlled study design, featuring a standardized study protocol. In addition, the use of a highly reliable Biodex system and the assessment of various validated clinical scores is also a strength of the study.

## Conclusion

The study supports the implementation of a modified ERAS concept for primary THA. The combination with a modified ERAS concept leads to a faster rehabilitation up to one-year postoperatively, reflected by significant better isokinetic strength. However, this finding is not reflected in the clinical outcome, showing no significant differences in validated clinical scores at any time point – possibly because clinical scores are not sensitive enough for small differences. Further studies are needed for long-term evaluation which should address the reported decline in physical function and health-related quality of life. Considering demographic trends, it will also be interesting to see to what extent an ERAS concept will be applicable to orthogeriatric patients, and whether this particular patient cohort can also benefit.

## Electronic supplementary material

Below is the link to the electronic supplementary material.

Supplementary Material 1

Supplementary Material 2

Supplementary Material 3

Supplementary Material 4

Supplementary Material 5

Supplementary Material 6

Supplementary Material 7

Supplementary Material 8

Supplementary Material 9

## Data Availability

On request, data is available at the authors’ institution.
