# Supplementary material for: Favourable mid-term isokinetic strength after primary THA combined with a modified enhanced recovery after surgery concept (ERAS) in a single blinded randomized controlled trial
Source: Arch Orthop Trauma Surg. 2024 Aug 6;144(8):3323–36. doi: 10.1007/s00402-024-05479-z (PMC11417056; doi:10.1007/s00402-024-05479-z)
Supplement: Supplementary file 5 — Supplementary Material 5 [file 402_2024_5479_MOESM5_ESM.docx]

# INDIVIDUAL CONFLICT OF INTEREST STATEMENT

***American Association of Hip and Knee Surgeons***

(Adopted from the American Academy of Orthopaedic Surgeons disclosure statement)

The following form **must be filled out completely and submitted by each author (example, 6 authors, 6 forms).**

**All items require a response. If there is no relevant disclosure for a given item, enter "*None*.”**

**Manuscript Title: Favorable mid-term isokinetic strength after primary THA combined with enhanced recovery after surgery (ERAS) in a single blinded randomized controlled trial.**

1. Royalties from a company or supplier (The following conflicts were disclosed)

**None**

2. Speakers bureau/paid presentations for a company or supplier (The following conflicts were disclosed)

**None**

3A. Paid employee for a company or supplier (The following conflicts were disclosed)

**None**

3B. Paid consultant for a company or supplier (The following conflicts were disclosed)

**None**

3C. Unpaid consultants for a company or supplier (The following conflicts were disclosed)

**De Puy (no contract)**

4. Stock or stock options in a company or supplier (The following conflicts were disclosed)

**None**

5. Research support from a company or supplier as a Principal Investigator (The following conflicts were disclosed)

**MSD, Novartis, De Puy, Otto Bock Foundation**

6. Other financial or material support from a company or supplier (The following conflicts were disclosed)

**support for symposias from De Puy, Orthotech, Ozo-zours, Fischer Fussfit, Urban & Kemmler**

7. Royalties, financial or material support from publishers (The following conflicts were disclosed)

**from Springer, Thieme, Trias, Zuckschwerdt, Rohwohlt for books**

8. Medical/Orthopaedic publications editorial/governing board (The following conflicts were disclosed)

**Springer, Thieme**

9. Board member/committee appointments for a society (The following conflicts were disclosed)

**DGOOC, DGOU (German orthopaedic societies)**

**Each author must sign AND print or type his/her name, date and submit a separate form**

In addition, one BLINDED Conflict of Interest form (no author names used) should be submitted per manuscript with all author disclosures.

Grifka, Joachim
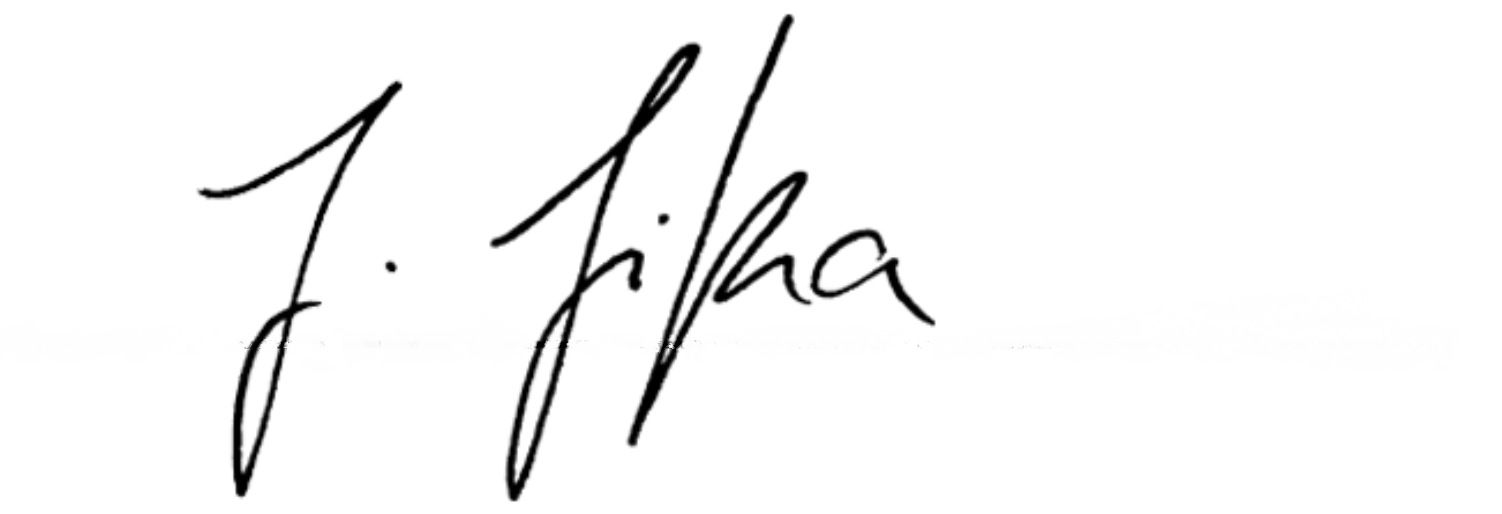
 03.12.2023

Author Name (Print or Type) Author Signature Date
